# The 2022 George E Palade Medal Lecture: Toxic Ca^2+^ signals in acinar, stellate and endogenous immune cells are important drivers of acute pancreatitis

**DOI:** 10.1016/j.pan.2022.12.010

**Published:** 2023-01-01

**Authors:** Ole H. Petersen

**Affiliations:** School of Biosciences, Sir Martin Evans Building, Cardiff University, Wales, CF10 3AX, UK

**Keywords:** Stimulus-secretion coupling, Stimulus-metabolism coupling, Necrosis, Macrophages, Imaging

## Abstract

In this account of the 2022 Palade Medal Lecture, an attempt is made to explain, as simply as possible, the most essential features of normal physiological control of pancreatic enzyme secretion, as they have emerged from more than 50 years of experimental work. On that basis, further studies on the mechanism by which acute pancreatitis is initiated are then described. Calcium ion signaling is crucially important for both the normal physiology of secretion control as well as for the development of acute pancreatitis. Although acinar cell processes have, rightly, been central to our understanding of pancreatic physiology and pathophysiology, attention is here drawn to the additional critical influence of calcium signaling events in stellate and immune cells in the acinar environment. These signals contribute significantly to the crucially important inflammatory response in acute pancreatitis.

## 1 Introduction

The George E Palade Prize and Medal is the highest honor awarded by the International Association of Pancreatology (IAP) to an individual who has made outstanding contributions to our understanding of the pancreas and pancreatic diseases. It is named after George E. Palade, who received the 1974 Nobel Prize in Physiology or Medicine. Palade worked on the exocrine pancreas and his greatest achievement was the discovery of the secretory pathway in the acinar cells and the process of exocytosis [1].

It was a great honour for me to receive the Palade Medal at the Joint meeting of the IAP with the Japan Pancreas Society in Kyoto on 7th July 2022. In this article, arising from the Palade Lecture I gave on that occasion, I shall not follow the autobiographical approach used so effectively by David Whitcomb in his Palade Lecture [2], simply because I have very recently published an autobiographical article after receiving Academia Europaea's Gold Medal [3]. Here, I shall try to describe – as briefly and simply as possible - the basic features of the physiological control of pancreatic enzyme secretion, as well as the pathophysiology of acute pancreatitis (AP). I shall describe some key experiments, illustrating how we have gained the knowledge that we have, because that understanding may be more important than the factual knowledge itself. We are currently drowning in data that do not provide us with useful knowledge [4] and we also have the problem that much of the knowledge we do possess collectively is not actually used by health professionals, largely because many do not know what is known [5]. My focus here will therefore be on what I think every pancreatologist should know.

## 2 Basic physiology of the control of enzyme secretion

Secretion of the pancreatic (pro)enzymes from the acinar cells occurs by the process of exocytosis and this was first described by George Palade [1]. Normal pancreatic enzyme secretion in response to a meal is mediated by the vagal nerve, releasing acetylcholine (ACh) from parasympathetic nerve endings in the neighborhood of acinar cells as well as by the hormone cholecystokinin (CCK). In both cases, secretion is initiated by a major change in intracellular Ca^2+^ movements [6]. By the time this article is published, it will be 50 years since we obtained this knowledge [7]. At that time, we also established that the pancreatic acinar cell, in contrast to what is known about endocrine and nerve cells, is electrically non-excitable. Depolarization of the acinar cell membrane, which can be achieved by exposing the cell to a solution with a high K^+^ concentration (for example, 50 mM, rather than the physiological 4.5 mM) does not elicit any change in Ca^2+^ movements and also does not evoke secretion [6,7].

**Fig. 1:**
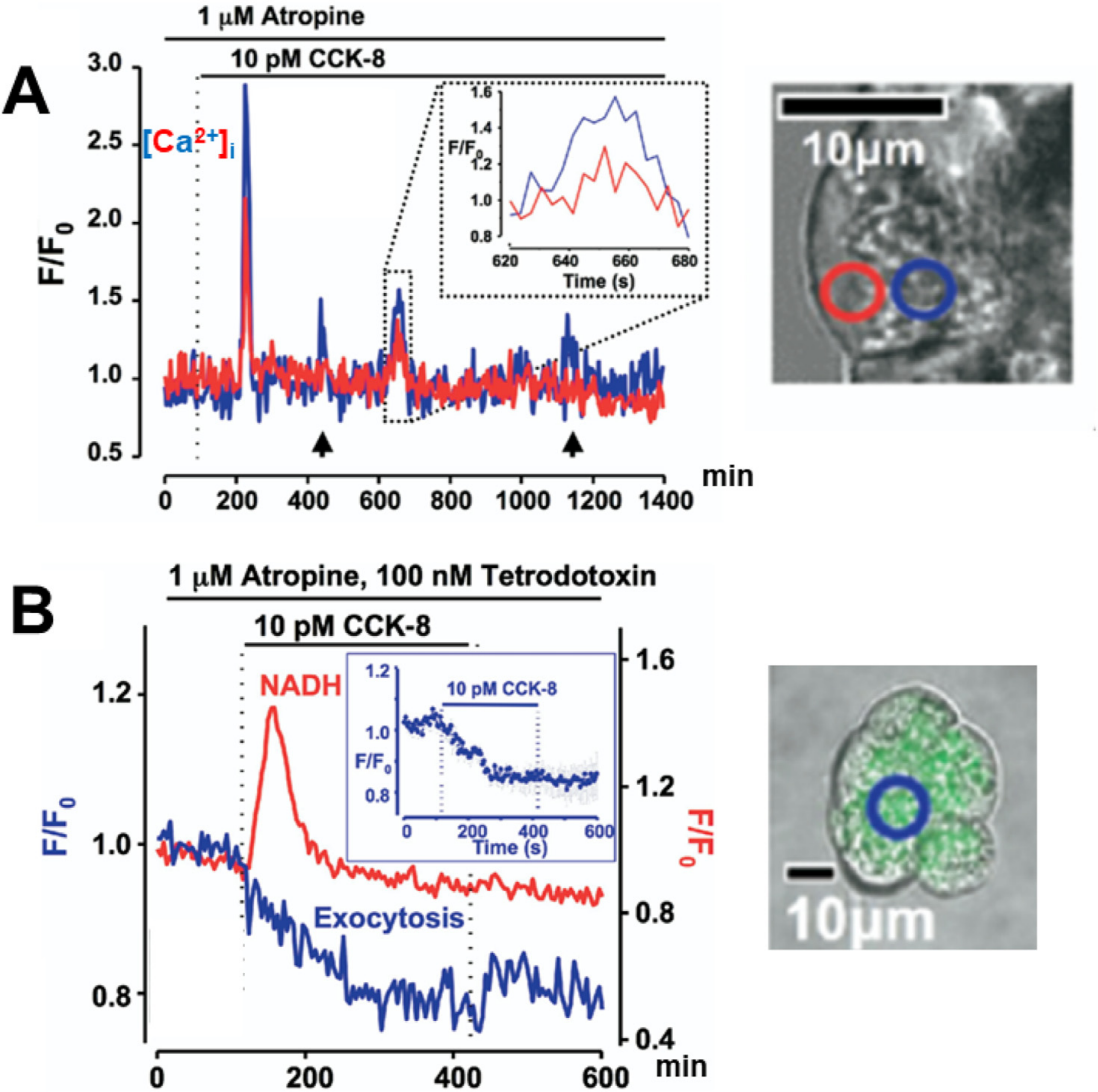
Stimulus-secretion and stimulus-metabolism coupling in human pancreatic acinar cells. **A** Intracellular Ca^2+^ signals evoked by 10 pM CCK-8 in an acinar cell from an isolated small acinar cell cluster (image to the right). The experiment was carried out in the presence of the muscarinic antagonist atropine. The blue trace represents [Ca^2+^]_i_ in the apical ZG-containing region, whereas the red trace is obtained from the basal area of the cell. The CCK-evoked Ca^2+^ spikes are mostly confined to the apical ZG-rich region of the acinar cell. **B** Effect of CCK-8 on NADH autofluorescence (red trace) and quinacrine fluorescence (blue trace) in an acinar cell from a small isolated acinar cell cluster (image to the right). The experiment was conducted in the presence of atropine and tetrodotoxin (to block any possible action potential generation in nerve endings that might have adhered to the acinar cluster). Quinacrine accumulates in acid organelles, such as the ZGs, and its disappearance therefore signals exocytotic secretion. Adapted from Murphy et al. *Gastroenterology* 2008 [10].

Many years later, it became possible to measure directly the ACh- or CCK-elicited changes in the cytosolic Ca^2+^ concentration ([Ca^2+^]_i_) [8]. Some further years later, we were also able to image the distribution of the rise in [Ca^2+^]_i_ elicited by ACh or CCK [9] and then, finally, we could demonstrate this in human pancreatic acinar cells (Fig. 1A). It turned out that physiological stimulation (for example with 10 pM CCK) did not cause a sustained elevation of [Ca^2+^]_i_ throughout the cell, but rather elicited repetitive short-lasting elevations, mostly confined to the apical zymogen granule (ZG)-containing region (Fig. 1A). A physiological CCK concentration (10 pM) also elicited exocytotic secretion as shown in Fig. 1B.

There has been controversy about the existence of functional CCK receptors on human pancreatic acinar cells [11], but it is clear from the type of data shown in Fig. 1, that CCK acts directly on human acinar cells and not via CCK-elicited release of ACh from nerve endings [10,12]. Our conclusion that the human acinar cells possess functional CCK receptors [10], as is the case for pancreatic acinar cells in all species investigated to date [13], has been independently confirmed by Gaisano's group in Toronto [12].

**Fig. 2:**
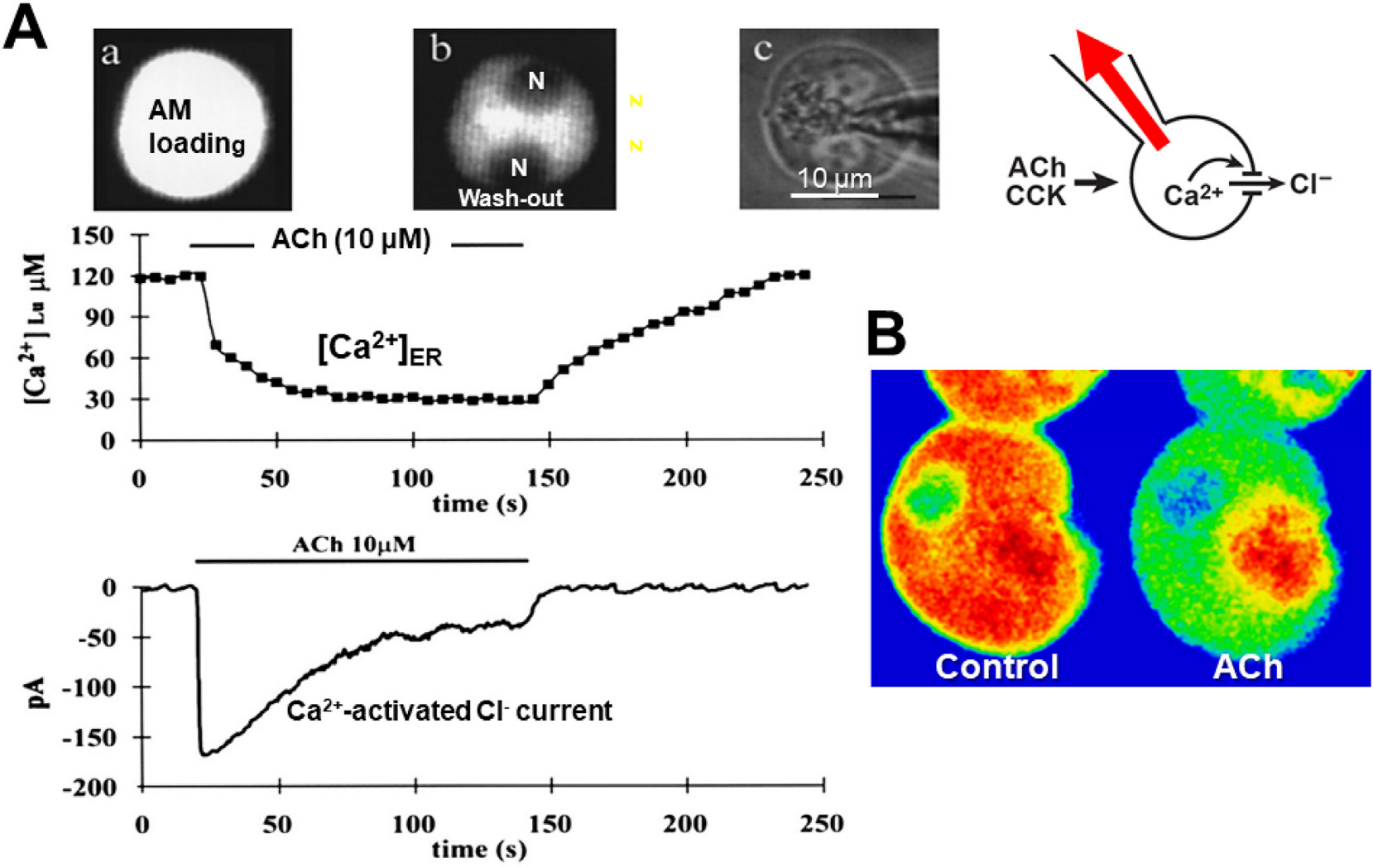
A supramaximal concentration of ACh empties the ER of Ca^2+^. **A** The two traces show the ACh-elicited changes in [Ca^2+^]_ER_ and Ca^2+^-dependent Cl^−^ current. This current monitors changes in [Ca^2+^]_i_ near the inner aspect of the apical membrane. A downward deflection therefore represents an increase in [Ca^2+^]_i_. The fluorescent low affinity Ca^2+^ indicator Mag-fura 2 was used to record changes in [Ca^2+^]_ER_. It was applied to the isolated acinar cell in its membrane permeant (AM) form and was therefore initially present throughout the cell (see image **a**). The indicator was subsequently washed out of the cytosol (**b**) via a patch clamp pipette in the whole cell recording mode (see sketch of recording configuration to the right) which was also used to measure the Ca^2+^-activated Cl^−^ current. The transmitted light mage of the cell is shown in (**c**). **B** Imaging of the [Ca^2+^] change in all stores of an isolated acinar cell before and after supramaximal ACh stimulation. Before ACh (Control) there is a uniform high level (red colour) of Ca^2+^ throughout the cell, except in the nucleus (which has the same (low) [Ca^2+^] as in the cytosol). Immediately after ACh stimulation, Ca^2+^ has disappeared from the whole of the baso-lateral ER-rich region (now green color), but not from the ZG-containing region. A is adapted from Mogami et al. *EMBO J* 1998 [14] and B from Park et al. *EMBO J* 2000 [15].

The early finding that the initial phase of ACh-elicited enzyme secretion does not depend on the presence of extracellular Ca^2+^, whereas sustained secretion is acutely dependent on external Ca^2+^, suggested that secretion is initiated by a Ca^2+^ signal generated by release of Ca^2+^ from internal stores [6,7,13]. This was directly shown in experiments in which changes in [Ca^2+^]_i_ were monitored by an endogenous Ca^2+^ sensor in the apical membrane of the acinar cell, namely the Ca^2+^-activated Cl^−^ channel [13], and the Ca^2+^ concentration in the ER ([Ca^2+^]_ER_) was assessed by a fluorescent probe trapped in the lumen of that organelle [14]. Fig. 2 shows an example with supra-physiological ACh stimulation evoking a sustained increase in [Ca^2+^]_i_ and an almost complete emptying of the ER Ca^2+^ store. In contrast, physiological stimulation, of the type shown in Fig. 1, only causes tiny reductions in [Ca^2+^]_ER_ in relation to each short-lasting Ca^2+^ spike [15].

ACh action on muscarinic type 3 receptors on the baso-lateral acinar cell membrane, generates the Ca^2+^ releasing intracellular messenger inositol 1,4,5-trisphosphate (IP_3_), which in turn opens Ca^2+^ release channels in the apical part of the endoplasmic reticulum (ER) [6,13]. Physiological CCK interaction with CCK1 receptors does not generate IP_3_, but acts primarily via generation of another Ca^2+^ releasing intracellular messenger, namely nicotinic acid adenine dinucleotide phosphate (NAADP) [13]. NAADP releases Ca^2+^ from acid stores as well as the ER, resulting in further release of Ca^2+^ from the ER, via the mechanism of Ca^2+^-induced Ca^2+^ release from ryanodine receptors, finally also recruiting IP_3_ receptors [13].

**Fig. 3:**
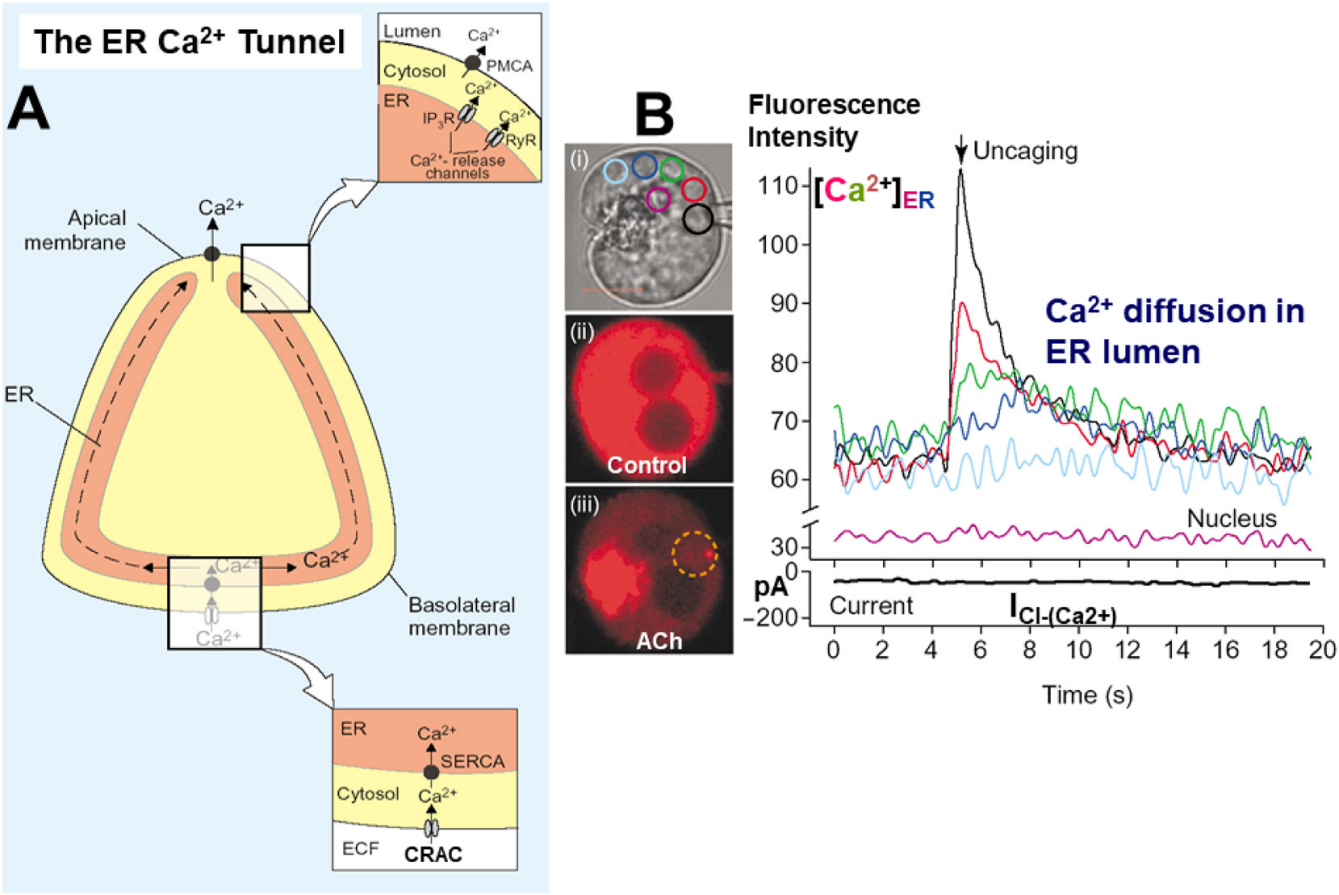
The Ca^2+^ tunnel function of the ER. **A** Schematic illustration of the model. At the top, Ca^2+^ release via IP_3_ receptors (IP_3_R) and ryanodine receptors (RyR) in the ER in the apical ZG-containing region and extrusion of Ca^2+^, via plasma membrane Ca^2+^-activated ATPase (PMCA) in the apical membrane. At the bottom, Ca^2+^ replenishment of the ER Ca^2+^ store via CRAC channels in the basal plasma membrane and subsequent uptake of Ca^2+^ across the ER membrane by Ca^2+^ pumps (SERCA – sarco-endoplasmic reticulum Ca^2+^-activated ATPase). Ca^2+^ can then diffuse in the lumen of the ER from the base to the apical region. **B** Experiment demonstrating directly the diffusion of Ca^2+^ in the ER lumen. (**i**) shows the transmitted light picture of the isolated acinar cell. The colored circles correspond to the respective colored [Ca^2+^]_ER_ traces in the main part of the figure. (**ii**) shows the uniform high [Ca^2+^] in the stores throughout the cell. After supramaximal ACh stimulation (**iii**) [Ca^2+^]_ER_ has been dramatically reduced (as already shown in Fig. 2) and uncaging of caged Ca^2+^ inside the ER in the apical region (iii) can now elicit a major rise in [Ca^2+^]_ER_. The traces to the right, show that the [Ca^2+^]_ER_ rise elicited by the local Ca^2+^ uncaging is highest in the apical region where the Ca^2+^ liberation occurs, but that this rise spreads relatively quickly (but of course with diminishing amplitude) to other regions of the cell, demonstrating directly the rapid movement of Ca^2+^ in the ER lumen. A is adapted from Petersen *Curr Biol* 2001 [16] and B from Park et al. *EMBO J* 2000 [15].

The bulk of the ER is located in the baso-lateral part of the acinar cell with only small and thin elements penetrating into the apical ZG-containing apical region [6,13]. It is therefore unsurprising that the major loss of Ca^2+^ elicited by supra-maximal stimulation occurs in the baso-lateral part of the cell (Fig. 2B). This, however, makes it difficult to understand why the cytosolic Ca^2+^ signals principally occur in the apical region and, even with supra-maximal stimulation, always are initiated in that region [6,13]. The explanation for this is the ER Ca^2+^ tunnel function (Fig. 3). The quantitatively most important Ca^2+^ release channel, the IP_3_ receptor, is localized in the ER terminals in the apical ZG-containing region. This could be shown in experiments where the whole of the cytosol was flooded with IP_3_ (or even a non-metabolizable IP_3_ analogue) causing Ca^2+^ spiking exclusively in the apical region [9]. The ER Ca^2+^ tunnel function [17] relies on the fact that the whole of the ER has one continuous lumen and that the Ca^2+^ binding capacity in the lumen of the ER is much lower than in the cytosol, so that Ca^2+^ diffuses more easily inside the ER than in the cytosol [6,13,15]. The relatively rapid diffusion of Ca^2+^ in the ER of the pancreatic acinar cells was demonstrated directly in experiments of the type shown in Fig. 3B.

The pancreatic acinar cell possesses a very sophisticated Ca^2+^ signaling system. Evolution has found an ingenious way to solve a difficult problem. Unlike other secretory cells, the pancreas needs to secrete large amounts of protein, as there is a need for substantial amounts of enzyme to deal with the digestion of food in the gut. This bulk secretion of proteins relies on massive exocytosis (partly compound exocytosis) and therefore the apical region - where the secretion process must occur through the apical membrane - needs to be tightly packed with ZGs. This leaves little room for the ER, which therefore mostly occupies the baso-lateral part of the cell. The Ca^2+^ signal initiating exocytosis obviously needs to happen near the apical membrane, whereas the bulk of the Ca^2+^ providing the source for the signal is at the base. The Ca^2+^ tunnel (Fig. 3) is Nature's clever solution.

During Ca^2+^ signaling, Ca^2+^ is lost from the cell because Ca^2+^ pumps, predominantly located in the apical membrane [6,13], are activated by the rise in [Ca^2+^]_i_ (Fig. 3). The ER Ca^2+^ store therefore needs to be replenished, otherwise the cell would run out of Ca^2+^. The replenishment occurs at the base through Ca^2+^ Release Activated Ca^2+^ (CRAC) channels (Fig. 3). In the pancreatic acinar cells, these extremely Ca^2+^-selective channels were for the first time characterized electro-physiologically in 2013 [18]. The CRAC channels are physiologically important, although the Ca^2+^ currents flowing through these pores under normal conditions are tiny. However, under pathological conditions, when the ER Ca^2+^ store is emptied completely, these channels become maximally activated and the resulting Ca^2+^ inflow is then responsible for the cellular Ca^2+^ overload that will eventually kill the cell by necrosis (see next section).

Secretion is energy consuming and, therefore, ATP generation must increase when the acinar cell is stimulated to secrete [13]. This happens in the mitochondria and Ca^2+^ plays the principal role by accelerating the Krebs cycle via the Ca^2+^-sensitive dehydrogenases [13]. In the pancreatic acinar cells, the mitochondria are mainly localized in a belt surrounding the granular region and they come very close to the apical membrane where secretion occurs [19]. The mitochondria sense the rise in [Ca^2+^]_i_ in the apical region and take up Ca^2+^ via the mitochondrial Ca^2+^ uniporter [13]. The resulting rise in the mitochondrial [Ca^2+^] then activates the already mentioned dehydrogenases, driving the ATP generation in the Krebs cycle. This can be monitored with good time resolution by recording the NAD(P)H autofluorescence (Fig. 1B). For each cytosolic Ca^2+^ spike there will be a NAD(P)H spike [13]. Thus, the cytosolic Ca^2+^ signals induced by physiological stimulation with ACh or CCK are crucial for both stimulus-secretion and stimulus-metabolism coupling [6,13].

## 3 Basic pathophysiology of acute pancreatitis

### 3.1 Acinar cells

Whereas Fig. 1A shows the result of stimulation with a physiological concentration of CCK, eliciting repetitive short-lasting local Ca^2+^ spikes, Fig. 2A illustrates the result of supramaximal stimulation, in this case with ACh, causing a sustained rise in [Ca^2+^]_i_ and complete emptying of the ER Ca^2+^ store. Such a sustained increase in [Ca^2+^]_i_ will only result in one short-lasting burst of ATP production [13]. The reason for the cessation of ATP generation is that overload of the mitochondria with Ca^2+^ evokes opening of a very large channel in the inner mitochondrial membrane, known as the mitochondrial permeability transition pore (MPTP). Opening of the MPTP results in a marked depolarization of the inner mitochondrial membrane. The normal very large electrical potential difference across the inner mitochondrial membrane is an essential requirement for ATP generation [13], so the collapse of this potential difference makes ATP generation impossible. In the absence of ATP, cell death can only occur by necrosis [13]. Therefore, when a sustained elevation of [Ca^2+^]_i_ is maintained for several minutes, it will cause necrotic cell death [13].

Although hyperstimulation, particularly with CCK or the amphibian analogue caerulein, has been used extensively as a model for inducing AP-like changes in the pancreas, it does not of course represent the real pathophysiology of AP. The major causes of AP are gallstone complications and alcohol abuse [20]. It therefore seems more relevant to study the effects of ethanol or bile acids, in order to understand the basic pathophysiology of AP.

**Fig. 4:**
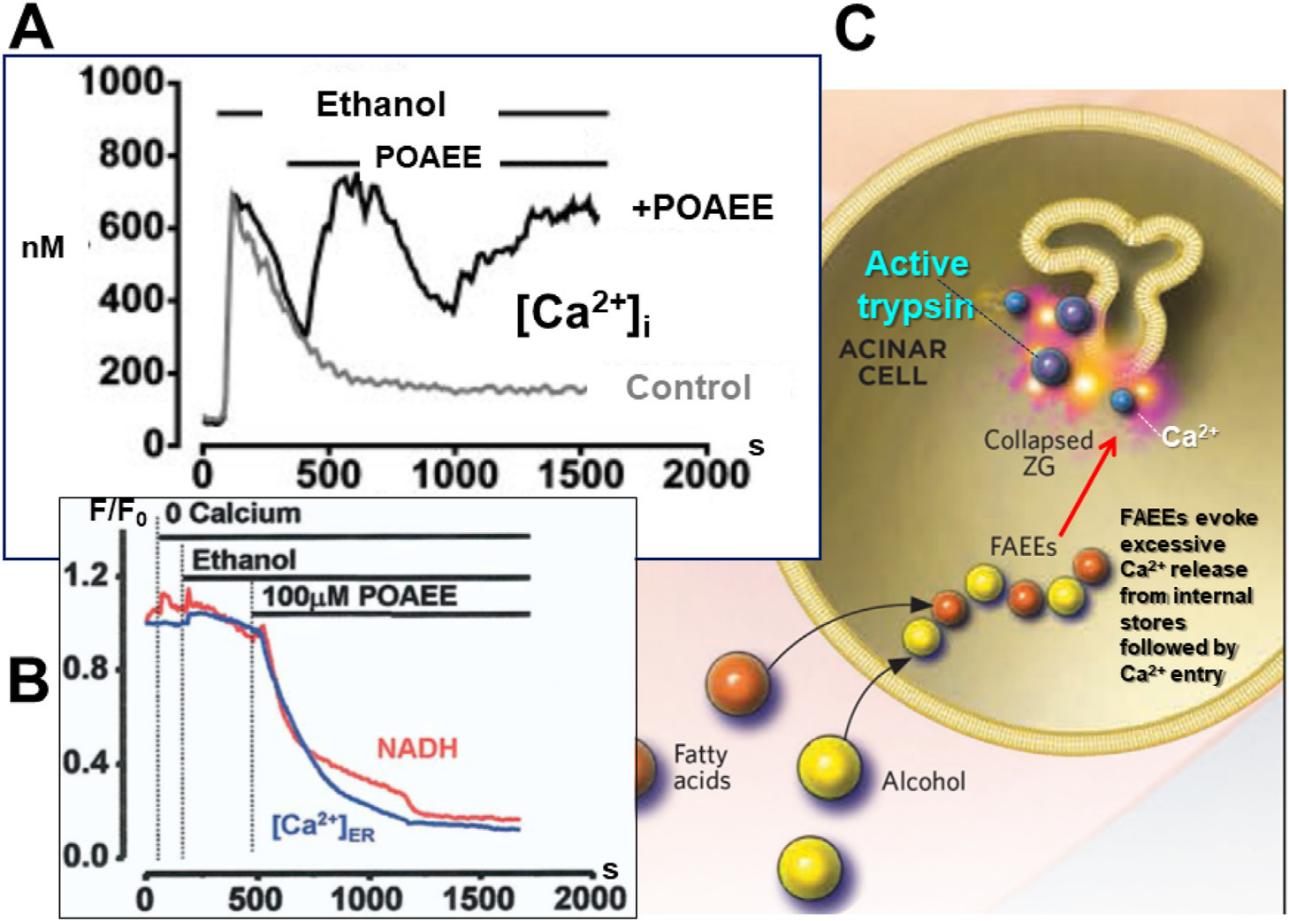
FAEEs are powerful releasers of Ca^2+^ from intracellular stores. **A** POAEE elicits a major and sustained rise in [Ca^2+^]_i_, whereas even an extremely high ethanol concentration (850 mM) only has a transient effect. **B** POAEE elicits a marked reduction in [Ca^2+^]_ER_ as well as a dramatic fall in NADH autofluorescence. **C** Schematic diagram illustrating the formation of FAEEs inside the acinar cell and also the subsequent destruction of the ZGs with release of active trypsin. A is adapted from Criddle et al. *PNAS* 2004 [23] and B from Criddle et al. *Gastroenterology* 2006 [24].

Unlike the situation in the liver, alcohol-elicited damage of pancreatic acinar cells is due to the non-oxidative generation of fatty acid ethyl esters (FAEEs) rather than alcohol itself or its oxidative metabolite acetaldehyde [21,22]. FAEEs are generated when ethanol combines with fatty acids (Fig. 4). The pancreas possesses particularly active FAEE synthases (carboxylester lipase) and is therefore capable of generating FAEEs in larger quantities than other tissues [21,25]. FAEEs are powerful releasers of Ca^2+^ from intracellular stores [24], whereas ethanol alone mostly has only very small effects, although occasionally evoking a short burst of intracellular Ca^2+^ liberation (Fig. 4).

The sustained [Ca^2+^]_i_ elevation evoked by palmitoleic acid ethyl ester (POAEE) (Fig. 4) depends completely on the presence of Ca^2+^ in the extracellular fluid and is driven by Ca^2+^ entry through the CRAC channels in the baso-lateral plasma membrane already mentioned (Fig. 3). The sustained elevated [Ca^2+^]_i_ (Fig. 4A) evokes intracellular trypsinogen activation [26], starting in the apical region [27] and this, together with the reduced ATP formation (the marked reduction of NADH autofluorescence is clearly seen in Fig. 4B), results in necrotic cell death [23,24]. Several bile acids produce similar effects to those described here for the action of FAEEs [28,29].

The mechanism by which the toxic Ca^2+^ signals evoked by FAEEs or bile acids are produced seems very similar to that induced by supra-maximal stimulation with ACh or CCK. The toxic Ca^2+^ signal generation depends mostly on functional IP_3_ receptors [13,24], but also to some extent on ryanodine receptors [29]. Some bile acids act on G protein – coupled receptors, generating IP_3_ [13], whereas FAEEs are more likely to act by modulating the sensitivity of the IP_3_ receptors to IP_3_, so that they open at the resting level of IP_3_ [13].

The excessive Ca^2+^ inflow through CRAC channels, evoked by complete emptying of the ER Ca^2+^ store, is the quantitatively dominant source of Ca^2+^ involved in generating the toxic cytosolic Ca^2+^ signals that ultimately destroy the acinar cells in AP. We know this because specific pharmacological inhibition of the opening of CRAC channels prevents all the deleterious effects of exposing the pancreas to alcohol and fatty acids, bile acids or toxic concentrations of ACh or CCK [18,30,31]. However, there are other channel types that can generate toxic Ca^2+^ signals and these can be activated by physical pressure. The pressure-sensitive channel Piezo-1 has been identified in pancreatic acinar cells [13,32]. When this channel opens, it allows a small inflow of Ca^2+^ which, apparently via phospholipase A2, opens TRPV4 channels that mediate a much larger Ca^2+^ inflow causing a toxic Ca^2+^ signal [32]. This mechanism may account for cases of AP that seem to have arisen from physical handling of the pancreas during surgery, as well as blockage of the main duct [13,32].

AP can occur as a side-effect of the treatment of acute lymphoblastic leukemia in children with asparaginase [33]. The asparaginase-induced AP is generated by a mechanism that is very similar to the ones that are responsible for alcohol- or bile-related AP. There is primary release of Ca^2+^ from the ER, which results in opening of CRAC channels, and pharmacological inhibition of CRAC channels is effective in preventing the development of AP during the action of asparaginase [34,35].

### 3.2 Stellate and immune cells

The acinar cell is by far the dominant cell type in the pancreas and is of course also the functionally most important, as it manufactures and secretes the enzymes needed for the digestion of food. The duct cells have long been recognized as essential, because they secrete the bicarbonate-rich fluid that helps to neutralize the acid gastric juice [6]. The neutral acinar and the alkaline ductal fluid secretion convey the secreted enzymes through the ductal system into the duodenum [6]. However, there are other cell types that should not be neglected.

**Fig. 5:**
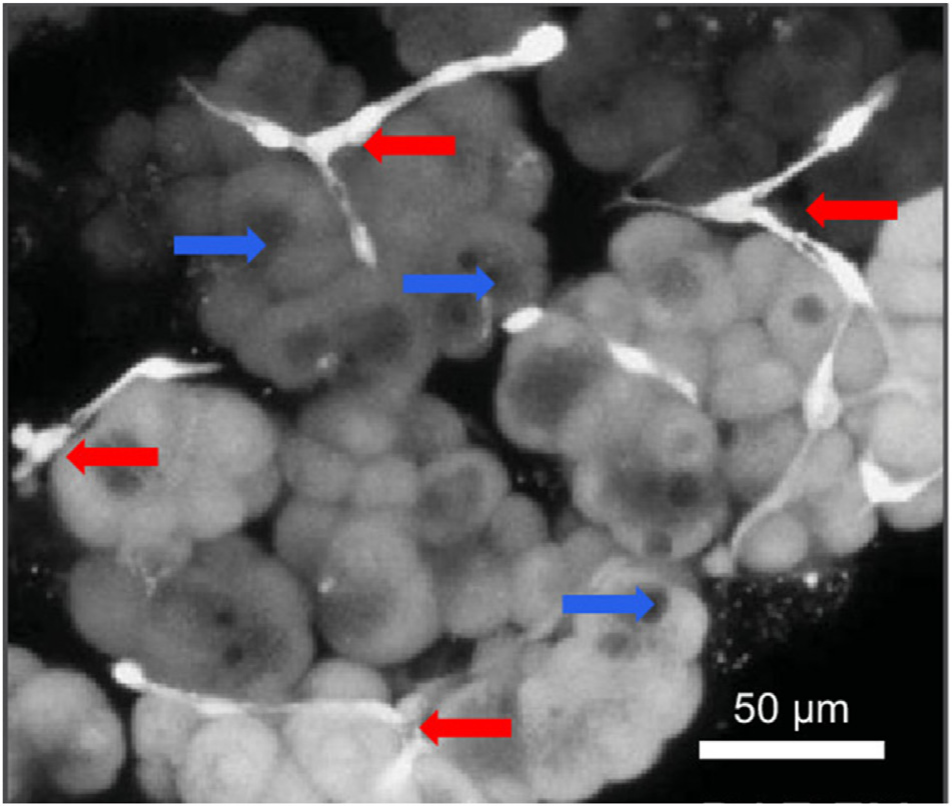
Live imaging of NO-sensitive fluorescence in pancreatic lobule stimulated with H_2_O_2_. The NO formation occurs in the stellate cells, which therefore light up (white) in this image. Adapted from Jakubowska et al. *Open Biology* 2016 [36].

The acinar environment contains stellate cells, which are difficult to see in transmitted light microscopy of the living pancreatic tissue. They can, however, easily be identified in the living tissue by fluorescence microscopy. The stellate cells take up various fluorescent probes more avidly than the acinar cells and thus can be made to ‘stand out’ (Fig. 5). Immune cells are more difficult to see in the normal living pancreas, as there are few of them and they can essentially only be identified by staining with specific antibodies at the end of functional studies [37]. The number of immune cells increases dramatically during the first days after induction of AP [37].

**Fig. 6:**
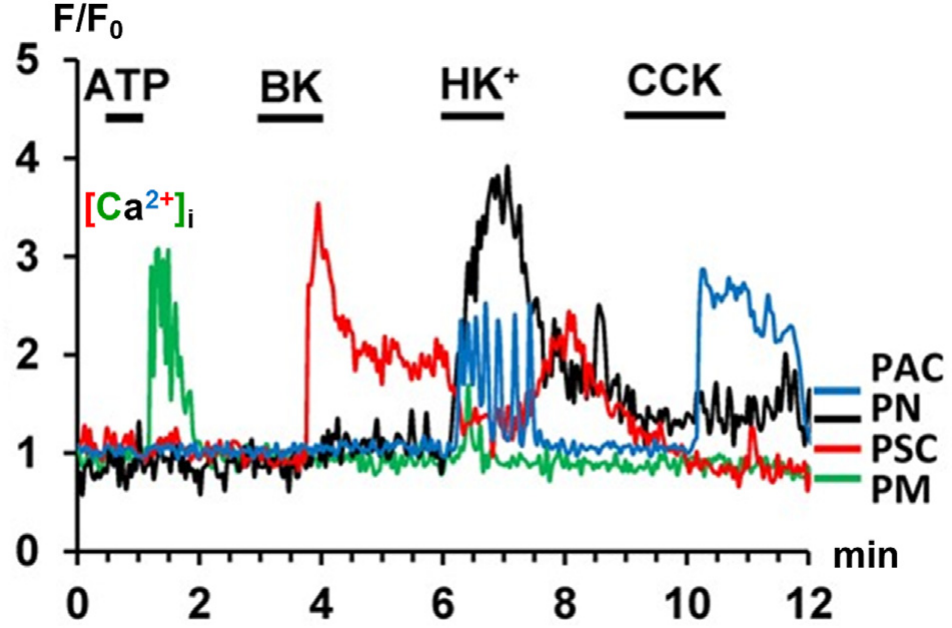
Simultaneous traces of [Ca^2+^]_i_ in different cell types recorded from a pancreatic lobule. The different colored traces represent measurements from a pancreatic acinar cell (PAC - blue), a pancreatic neuron (PN – black), a stellate cell (PSC – red) and a macrophage (PM – green). See text for further explanation. Adapted from Gryshchenko et al. *Function* 2021 [37].

We are now able to simultaneously monitor changes in [Ca^2+^]_i_ in several different pancreatic cell types in quasi-intact pancreatic lobules [[37], [38], [39]]. This is a useful method to obtain an overview of the response pattern to various stimuli in the different cells. Fig. 6 shows an example of such an experiment. In a cell that was identified as a macrophage, by post-functional specific antibody staining [37], ATP evoked a cytosolic Ca^2+^ signal (green trace), whereas none of the other cell types responded. Thereafter, the pro-inflammatory nonapeptide bradykinin (BK) evoked a prolonged rise in [Ca^2+^]_i_ in a stellate cell (red trace), but none of the other cells reacted to this stimulus. When the [K^+^] in the bath solution was increased 10-fold (from 5 to 50 mM), there was a dramatic rise in [Ca^2+^]_i_ in a nerve cell (black trace), which was quickly followed by a series of repetitive Ca^2+^ spikes in an acinar cell (blue trace). Finally, CCK application evoked a rise in [Ca^2+^]_i_ in the acinar cell (blue trace), whereas none of the other cells reacted. Further tests revealed that the acinar Ca^2+^ signals elicited by a high-K^+^ stimulus were mediated by release of ACh from nerve endings near the acinar cells, because atropine blocked the rise in [Ca^2+^]_i_ in the acinar cells, but not in the nerve cells [39].

BK is the principal agent evoking Ca^2+^ signals in stellate cells. Any increase in the BK concentration above the normal resting plasma level will evoke Ca^2+^ signals in these cells [13,38,39]. In AP, the plasma BK level is increased, so this will cause Ca^2+^ signal generation in the stellate cells, which will induce secretion of inflammatory agents [13]. The stellate cell Ca^2+^ signals also activate the Ca^2+^-sensitive enzyme Nitric Oxide (NO) synthase resulting in NO generation (Fig. 5). It would appear, that NO, perhaps indirectly, has a deleterious effect on the neighboring acinar cells, as pharmacological inhibition of NO synthase provides considerable protection against the necrosis evoked by, for example, bile acids [36].

The macrophages have metabotropic purinergic receptors (P2Y_1_ and P2Y_13_) and react equally well to ATP and ADP. Necrotic acinar cells release ADP, which in turn can activate the immune cells, resulting in cytokine secretion [13,37]. A minority (∼40%) of the endogenous pancreatic macrophages also displayed Ca^2+^ signals in response to BK [37]. Ca^2+^ signal generation in both stellate and immune cells occurs via much the same mechanism as in the acinar cells. Stimulation with BK in the stellate cells, and ADP in the macrophages, results in IP_3_ generation, which primarily releases Ca^2+^ from the ER. This is then followed by opening of CRAC channels in the plasma membrane [13,37,38]. It follows that pharmacological inhibition of CRAC channels will reduce Ca^2+^ signaling in all three cell types, namely the acinar, stellate and immune cells [13,37,38].

## 4 A new model concept for the initiation of acute pancreatitis

**Fig. 7:**
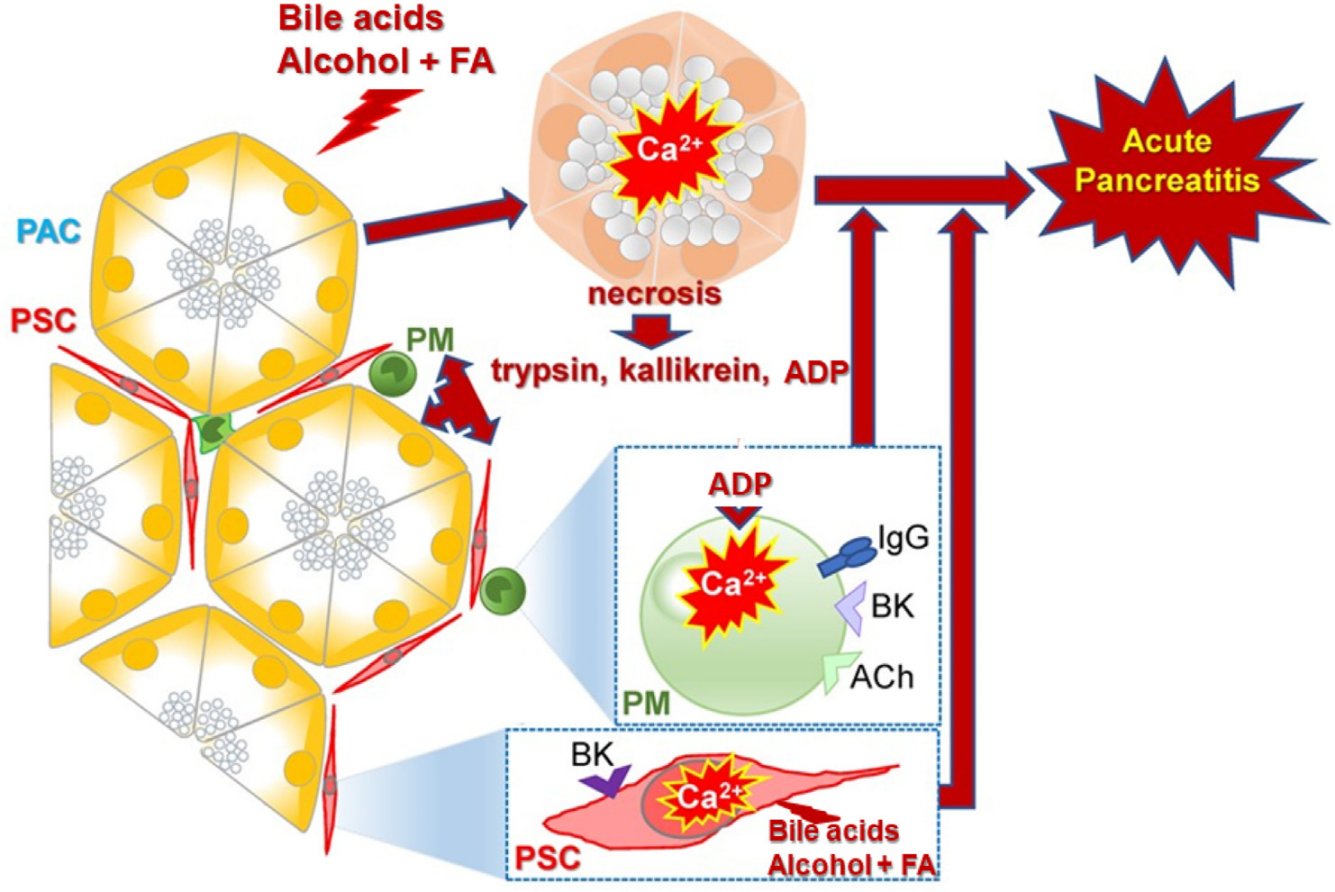
Schematic illustration of the various processes involved in the development of AP. Adapted from Gryshchenko et al. *Function* 2021 [37].

Our insights concerning the mechanism by which AP is initiated, derived from experiments of the type described in this article, are summarized in Fig. 7. The general model proposed in our recent review article [13], emphasized a sequence in which the primary insult, alcohol + fatty acids or bile acids, acts on acinar cells to generate toxic Ca^2+^ signals leading to necrosis. This would release a number of proteases, including trypsin and kallikrein, into the acinar environment. Kallikrein would release BK from bradykininogen and this small peptide would then act on stellate cells to secrete inflammatory agents. ADP, released from necrotic acinar cells, would act on the immune cells to further increase the inflammatory response. However, recent data now indicate that FAEEs can also directly elicit large Ca^2+^ signals in the stellate cells [40]. Some bile acids are also capable of evoking Ca^2+^ signals directly in the stellate cells [41]. These data are important, because they indicate the possibility that primary insults of the stellate cells could be the prime driver of the inflammatory response, which is by far the most significant danger in AP [20].

Trypsinogen activation in the acinar cells is clearly a crucial driver of acinar necrosis in AP [42,43], particularly in conjunction with the reduction in ATP synthesis [24]. However, an important study by Saluja, Garg and their collaborators [44] showed that absence of trypsinogen activation, due to deletion of a trypsinogen gene, although protecting against acinar necrosis, did not reduce the inflammatory response in AP [44]. In this context, it is interesting that the effects of alcohol + fatty acids [40], as well as some bile acids [41], on the stellate cells could potentially induce inflammation directly. This point is further emphasized by recent data showing that the spike protein of SARS-CoV-2 acts directly on the stellate cells to elicit Ca^2+^ signals and interleukin secretion, which then in turn activates macrophages [45]. The stellate cell may thus have a central role in generating the inflammatory response in AP.

Although Fig. 7 does not include duct cells, this should not be taken to indicate that these cells do not play any role in the development of AP. The ductal bicarbonate-rich fluid secretion is clearly important for transporting the digestive (pro)enzymes into the duodenum and inhibition of ductal fluid secretion, which occurs in AP, will therefore exacerbate autodigestion because the enzymes stay longer in the duct system [46]. There are also important interactions between acinar and duct cells [46] that are outside the scope of this article.

## 5 Future perspectives and opportunities

The basic science work reviewed here has identified several targets for potential pharmacological therapy in AP. The CRAC channels, present in all the 3 cell types discussed here, are obvious targets and this is currently being explored in clinical trials [13,47]. BK type 2 receptors, principally in the stellate cells, but also present in some immune cells, are also potentially attractive targets, as antagonists would break a vicious necrotic amplification loop, involving stellate and acinar cells [13]. There are also indications that AP may, in part, be a metabolic disease, as glucose metabolism is inhibited due to partial blockage of the initial hexokinase-mediated generation of glucose-6-phosphate [35]. This step can be by-passed by galactose [13] and this has been shown to protect against cell damage in mouse models of AP [35].
